# Human Papillomavirus 16 Non-European Variants Are Preferentially Associated with High-Grade Cervical Lesions

**DOI:** 10.1371/journal.pone.0100746

**Published:** 2014-07-01

**Authors:** Luciana Bueno Freitas, Zigui Chen, Elaine Freire Muqui, Neide Aparecida Tosato Boldrini, Angélica Espinosa Miranda, Liliana Cruz Spano, Robert D. Burk

**Affiliations:** 1 Post-Graduate Program in Infectious Diseases, Federal University of Espírito Santo, Vitória, Brazil; 2 Department of Pediatrics, Albert Einstein College of Medicine, Bronx, New York, United States of America; 3 Department of Pathology, Center of Health Sciences, Federal University of Espírito Santo, Vitória, Brazil; 4 Departments of Pediatrics, Microbiology and Addresses Immunology, Epidemiology and Population Health, and Obstetrics, Gynecology and Woman’s Health, Albert Einstein College of Medicine, Bronx, New York, United States of America; Institut National de la Santé et de la Recherche Médicale, France

## Abstract

HPV16 accounts for 50–70% of cervical cancer cases worldwide. Characterization of HPV16 variants previously indicated that they differ in risks for viral persistence, progression to cervical precancer and malignant cancer. The aim of this study was to examine the association of severity of disease with HPV16 variants identified in specimens (n = 281) obtained from a Cervical Pathology and Colposcopy outpatient clinic in the University Hospital of Espírito Santo State, Southeastern Brazil, from April 2010 to November 2011. All cytologic and histologic diagnoses were determined prior to definitive treatment. The DNA was isolated using QIAamp DNA Mini Kit and HPV was detected by amplification with PGMY09/11 primers and positive samples were genotyped by RFLP analyses and reverse line blot. The genomes of the HPV16 positive samples were sequenced, from which variant lineages were determined. Chi^2^ statistics was performed to test the association of HPV16 variants between case and control groups. The prevalence of HR-HPV types in <CIN1, CIN2 and CIN3+ were 33.7%, 84.4% and 91.6%, respectively. Thirty-eight of 49 (78%) HPV16 positive samples yielded HPV16 sequence information; of which, 32 complete genomes were sequenced and an additional 6 samples were partially sequenced. Phylogenetic analysis and patterns of variations identified 65.8% (n = 25) as HPV16 European (E) and 34.2% (n = 13) as non-European (NE) variants. Classification of disease into CIN3+ vs. <CIN3 indicated that NE types were associated with high-grade disease with an OR = 4.6 (1.07–20.2, p = 0.05). The association of HPV16 NE variants with an increased risk of CIN3+ is consistent with an HPV16 genetically determined enhanced oncogenicity. The prevalence of genetic variants of HPV16 is distributed across different geographical areas and with recent population admixture, only empiric data will provide information on the highest risk HPV16 variants within a given population.

## Introduction

Human Papillomaviruses (HPVs) are double stranded DNA viruses with an 8 Kb episomal genome. The organization of the genome is divided into three functional regions: an upstream regulatory region (URR) that regulates the transcriptional and replication events; an early region that expresses the non-structural proteins (e.g., E1, E2, E4, E5, E6, E7), and a late region that encodes the structural proteins L1 and L2 [1].

HPV belongs to the *Papillomaviridae* family, which includes more than 170 different types of characterized and designated viruses [2]–[4] (for review see www.hpvcenter.se/html/refclones.html). The papillomavirus members are classified into types based on the DNA sequence of the ORF of the major capsid protein, L1. A new viral type is assigned if the complete genome is cloned and the difference in the L1 nucleotide sequence is at least 10% different than all other classified HPV types [2], [3]. Around 40 genotypes can be identified in the anogenital region, and are associated with warts, cervical intraepithelial neoplasia (CIN) and cervical cancer (CC) [1], [5]–[8].

According to the prevalence of specific HPV DNA types in cases of cervical cancers, the anogenital HPVs have been classified into low and high risk types [9]–[13]. Although the etiology of CC is well established, HPV infection alone is not sufficient for the cancer’s development. Additional risk factors are in part related to the progression of HPV infections to carcinoma *in situ* and cancer including smoking, hormonal contraceptive use, multiple pregnancies and possibly other factors [14]–[18]. Factors related to the virus also contribute to progression of the infection to cancer, such as HPV type involved in the infection, viral variants, persistence and viral load [5], [10], [19], [20]. Of the high-risk HPV (HR-HPV) types associated with cervical cancer, HPV16 is the most prevalent and it is found in approximately half of all cancers [10], [12], [21]. Within the PV research community, isolates of the same HPV type are referred to as variants or subtypes when the nucleotide sequences of the L1 ORF differ by less than 10% [22]. Significant differences in pathogenicity exist between variants within a single HPV genotype and have been elucidated most clearly for HPV16, whose variants differ in their association with CC, viral persistence and frequency of recurrence of cervical disease [22], [23]–[35].

The description and understanding of HPV genome variants is an important area for molecular pathogenesis and for the development of molecular diagnostics for HPV, vaccines and other therapeutic approaches to control and/or eliminate virus-induced diseases. The tumorigenicity of the HPV variants could be different among geographical areas because of population history and host genetics, such as the difference in the distribution of HLA in the population [27], [36]. However, few studies from Brazil have reported on the prevalence of HPV DNA in the genital tract and natural history of infections, especially associating phylogenetic variants in the population with the severity of intraepithelial lesions [37]–[43].

As has been demonstrated by biochemical and biological differences of HPV16 variants and their oncogenic potential changes [22], [37], [44], [45], the description of oncogenic variants of HPV types should contribute to understanding the genetic determinants related to the development of high-grade lesions and the incidence of CC in specific populations.

## Materials and Methods

Cervical smears (n = 281) were obtained during gynecological visits at the Colposcopy outpatient clinic in the University Hospital “Cassiano Antonio Moraes” (HUCAM) in Vitória, Southeastern Brazil, from April 2010 to November 2011. This research obtained approval by the Ethical Research Council of the Center of Health Sciences of the Federal University of Espírito Santo, Brazil, in November 2009; all the participants signed an informed consent.

All cytologic and histologic diagnoses were determined prior to definitive treatment and were classified as <CIN3 (normal, CIN 1, 2), n = 257, used as the comparison or control group, and CIN3+ (CIN3 or worse), n = 24, the case group for this study. The classification in control (<CIN3) or case (CIN3+) group was used in the context of the HPV16 variants results. The DNA was isolated using QIAamp DNA Mini Kit (Qiagen, Valencia, CA) according to the manufacturer’s instructions. The HPV DNA was detected by amplification with PGMY09/11 primers [46]. HPV positive samples were genotyped by Restriction Fragment Length Polymorphism (RFLP) from gel analyses [47] and by Reverse Line Blot Hybridization (RLB) [48]. The genomes of the HPV16 positive samples were further characterized for the current study by amplifying the complete genome (∼8 Kb) using nested PCR of 3 or 4 overlapping fragments employing type-specific primer sets (available from authors) as described [49]. For overlapping PCR, an equal mixture of Ampli*Taq* Gold DNA polymerase (Applied Biosystems, Carlsbad, CA) and Platinum *Taq* DNA Polymerase (Invitrogen, Carlsbad, CA) were utilized as previously described [50].

The PCR product sizes were confirmed by gel electrophoresis, purified using the QuickStep 2 PCR Purification kit (Edge BioSystems, Gaithersburg, MD) or QIAquick Gel Extraction kit (Qiagen, Valencia, CA). The amplified fragments were directly sequenced on an ABI Prism Model 377 automated sequencer (Perkin-Elmer Applied Biosystems) in the Einstein DNA Sequencing Core Facility (Bronx, NY). The sequences of the fragments obtained were assembled using Geneious v6.1.6 [51], and aligned using MAFFT v6.903b [52], together with HPV16 reference sequences of each sublineage (Table S1). The construction of the phylogenetic tree inferred from the aligned sequences was performed using the software PhyML [53]. Chi^2^ statistics was performed to test the association of HPV16 variants between case and control groups.

## Results

The median age of participating women was 38.7 years (SD 10.97). Out of 281 samples, 56% (157/281) were positive for HPV DNA. All of these positive samples were genotyped by RFLP and RLB and HR-HPV was found in 124 samples (79%, 124/157), from which 32.3% (49/124) were positive for HPV16. Based on cytology results, HR-HPV types were detected in 33.7% (76/225) from <CIN1, in 84.4% (27/32) from CIN2 and in 91.6% (22/24) from CIN3+. HPV16 was found in 14% (35/257) and 58% (14/24) of the samples classified as <CIN3 and CIN3+, respectively (p<0.001).

**Figure 1:**
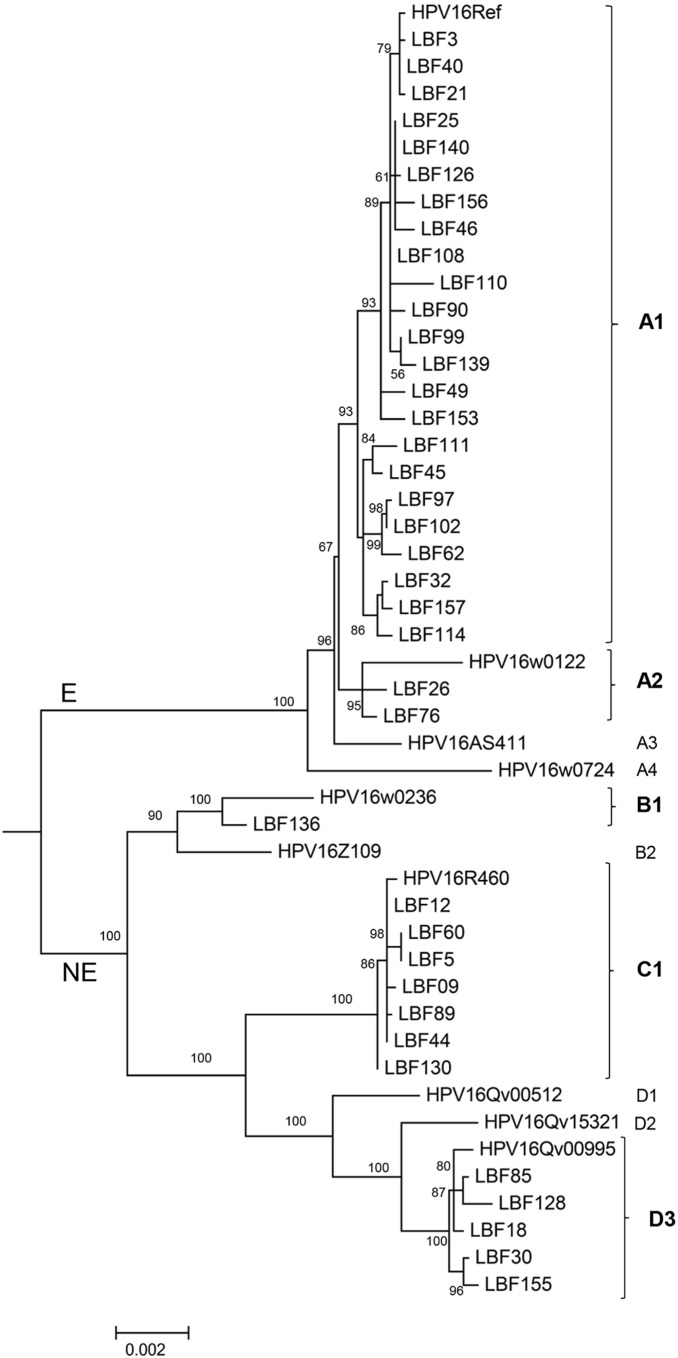
Tree topology. Phylogenetic tree was inferred from global alignment of complete and partial genome nucleotide sequences. Distinct variant lineages (i.e., termed A, B, and C) are classified according to the topology and nucleotide sequence differences from >1% to <10%; distinct sublineages (e.g., termed A1 and A2) were also inferred from the tree topology and nucleotide sequence differences in the >0.5% to <1% range [22].

The HPV16 complete genome was characterized for 32 samples and partial genome information was obtained for 6 using HPV16 specific overlapping PCR [49]. The nucleotide sequences obtained for all 38 samples were compared with the HPV16 prototype of each HPV16 variant lineage and sublineage and based on the phylogeny, variants were assigned to a specific lineage (Figure 1). Phylogenetic analysis classified 65.8% of the samples as HPV16 European (E, A lineage) (n = 25) and 34.2% as non-European (NE, lineages B, C, and D) (n = 13) variants. Isolates of the E group/A lineage were further classified to sublineages A1 (60.5%, 23/38) and A2 (5.3%, 2/38), and isolates from the NE group/lineages B/C/D sorted to sublineages B1 (Af-1) (2.6%, 1/38), C1 (Af-2) (18.4%, 7/38), and D3 (AA1) (13.2%, 5/38) (Figure 1). Taken together, samples containing HPV16 NE variants were associated with high-grade disease (CIN3+) with an OR = 4.6 (95% CI: 1.07–20.2; p = 0.05) compared to those with HPV16 E variants (Table 1). The nucleotide differences amongst the sequenced genomes are shown in Figure S1. The T/G variation at nucleotide 350 (gene *E6*) was not associated with CIN3+ (Figure S1).

**Table 1:** HPV16 variant distribution by diagnostic category.

	HPV16		
Cytology	E	NE	Total
<CIN3	20	06	26
CIN3+	05	07	12
Total	25	13	38

<CIN3: control group, comprising the normal and cervical intraepithelial neoplasia (CIN) grades 1 and 2;

CIN3+: case group, comprising the samples from CIN 3 or worse (cervical cancer in situ or invasive);

E: HPV16 European variant; NE: HPV16 non-European variant.

## Discussion

Based on complete and partial genome analyses, this study described the association of non-European HPV16 variants lineages/sublineages in women from Vitoria Brazil with CIN3+ cervical lesions. There is a proposed hypothesis about the differences in pathogenicity existing among variants of a single HPV genotype [22], [28]. Studies have demonstrated that HPV16 variants differ in their association with CC [24], [25], [28], [54]–[56] and viral persistence [23], [26], [29], [30], [32].

The prevalence of molecular variants from each branch in different geographical areas varies significantly and correlates with the intrinsic admixture level of each population [49], [57], [58]. An increased risk of developing high-grade CIN/cancer was observed in association of HPV16 non-European variants in several studies in the world [22], [32], [45], [59]–[63]. In addition, a number of reports in Brazil have described the presence of HPV16 variants in cervical samples and/or in association with different grades of lesions [37]–[43].

All sequenced HPV16 genomes showed at least one specific nucleotide variation compared to the HPV16-E prototype sequence. Regarding HPV16 sublineages, defined as containing 0.5–1% of nucleotide variations, the described population had a relatively heterogeneous set of HPV16 variants found in the following frequency order: A1>Af-2>AA>A2>Af-1. A study conducted with cervical samples from Central Brazil, identified AA variants as the second most common lineage of HPV16, with samples from the E branch being most common [38]. It was described AA/NA variants in cases from cervical cancer in South/Central America in association with high grade cervical lesions which might be related to differences in transcriptional activity, that were higher than E isolate variants [60]. This feature might be one possible explanation for the association between the NE variants in CIN3+ cases in the present study. The HPV16 C lineage (Af-2) was the second most common variants in the current report, but due to the limited sample size it is not possible to ascribe specific risks to sublineages, nevertheless 3/12 cases had C lineage isolates vs. 4/26 controls; and 3/12 cases had D lineage isolates vs. 2/26 controls. Studies conducted in Central or Southeastern Brazil have not found the HPV16 Af variants or it was identified infrequently [37], [38]; which, has been detected relatively commonly in Argentinean Indians [64]. The difference in geographic distribution of HPV16 variants is likely related to the population history of the region reflecting the influx of Europeans, Indian/native populations and people of African descent. Similar results of geographic origins have been reported and were the basis to suggest that HPV16 variants reflect the relatively recent human migration patterns [65].

In the present study it was found that HPV16 NE variants were significantly associated with CIN3 or worse lesions. Another study, with women from Northern Brazil found NE variants associated with high-grade cervical lesions [42]. However, HPV16 NE variants were detected at similar frequencies in low grade lesions (6/41, 14.6%) and in high grade cases (4/41, 9.7%) in a study conducted in São Paulo, also in Southeastern Brazil [39] and HPV16 NE and E variants have been detected at similar frequencies among the cytological finds (atypical squamous or glandular cells of undetermined significance, cytological alterations suggesting HPV infection, CIN, squamous cell carcinoma, and adenocarcinoma) in women from Central Brazil [38], not supporting a role for NE HPV16 variants as at increased risk for CC. Nevertheless, there is other evidence that HPV16 NE variants have elevated risks for CIN3 and cancer, although much of the effect was related to the increased risk with the AA (D) lineage [25], [56], [66], and there appears to be geographic complexity [58]. There are also reports that indicate the HPV16 AA (D) lineage compared to the E (A) lineage is disproportionately (4–35 fold increased) associated with adenocarcinoma (AdCa) vs. squamous cell carcinoma (SCC) [25], [56], [67], [68]. The differences in studies probably relates to the level of admixture of different HPV16 variants within a population.

The nucleotide substitutions in the samples from the lineage A have not shown any association with the cases, corroborating the negative association of the E variants with high-grade lesions. On the other hand, the SNPs detected along the complete genome from the NE variants are highly correlated and it is difficult to identify specific SNPs that might have unique pathologic consequences. The frequency of the Af-2 variants and AA in the NE branch could reflect the admixture of the population studied. The substitutions in the URR region can affect the transcription binding sites including activator protein 1 (AP1), nuclear factor 1 (NF1), octamer-binding protein 1 (Oct1), glucocorticoid/progesterone response element (GRE), specificity protein 1 (SP1), transcription enhancer factor 1 (TEF1), and yin yang 1 (YY1) [69], [70]. The substitution observed in the NE samples (A7458T), but not in the E samples, can affect the NF1 binding site and the ACCN_6_GGT sequence recognized by the E2 protein in the URR region [71] which could be also related to the oncogenicity. The nucleotide alterations at the position of the transcriptional factors binding site (TFBS) could reflect in the HPV replication, and consequently in the malignancy induction in the cervix. Some point mutation could be observed at the binding sites TEF-1 (G7193T, C7689A), GRE-1 (A7458T, A7485C, G7489A) and YY1 (G7521A, C7786T, G7826A, A7837C, A7839G). One of the changes, as C7689A (TEF1 site), was found in NE samples significantly associated with cases. In a previous study, Kämmer et al. [69] observed that nucleotide variations, although not inside the TFBS, but located adjacent to them, were probably responsible for the increase of 3.9-fold on the transcriptional activity of P97 promoter. Accordingly, besides the mutations located in the binding sites it was found in our study some adjacent nucleotide alterations that could alter the function of the mentioned transcriptional factors. HPV isolates from cervical cancer show frequent point mutations or deletions at YY1 binding sites on the LCR, which may be responsible for the increase of the transcriptional activity observed for these isolates [72], [73]. However with the small numbers of cases, the present study cannot confirm the relation of the TFBS with the grades of cervical lesions.

Increasing studies performed around the world, including Brazil, indicate the relationship between HPV16 variants and higher oncogenic risk is complex [74], [75], thus a well-planned epidemiological study is needed to evaluate HPV16 single nucleotide polymorphisms and oncogenic risk. For example, there is a relatively common SNP with the E6 ORF (T350G), which is a non-synonymous change resulting in an amino acid variation (L83V). This variation/mutation might be related to higher oncogenic potential [23], [24], [76], [77], or not [33], [78], [79]; in the current study it was not found to be associated with increased risk. It has been suggested that this mutation is associated with CC in a heterogenic form by world region [58]. The E variants harboring the 350T were significantly associated with the cancer risk in comparison with those with the mutation 350G in samples from Europe/Central Asia and East Asia, while the opposite was true in South/Central America [80]. A similar strong association of EUR-350G with cervical cancer has been observed in previous studies from Argentina [81] and Morocco [45].

Moreover, miss-sense nucleotide mutations theoretically could alter the epitopes targeted by the current HPV vaccine [82]. The investigation of circulating HPV variants is important not just in the light of the viral and concomitant viral evolution, but also in understanding the pathogenesis of HPV in malignant lesions. It will also be important to follow vaccinated populations to establish whether the oncogenic HPV genomes might have greater mutational variability and/or ability to mutate than has currently been documented. It is not thought that the oncogenic HPV types will be able to evade the current vaccines, but only empirical evidence will allow this question to be addressed in the decades to come.

The association of HPV16 non-European variants with CIN3+ is consistent with a genetically determined enhanced oncogenic potential of the NE HPV16. These observations suggest that determination of HPV16 variant lineage has clinical implications. The complete genome sequencing has the goal of allowing the genetics of HPV16 to inform us about differences in HPV biology, and permit continued improvements in phylogenic classification of subgroups with even higher oncogenic risks.

The prevalence of genetic variants of HPV16 is distributed across different geographical areas and with recent population admixture, Brazil is an ideal location to study the biology and clinical importance of HPV variants.

## Supporting Information

Figure S1 **Nucleotide variations compared to the HPV16 reference sequence.** The nucleotide positions of detected variations are shown across the top and are indicated by the corresponding nucleotide letter. The absence of variations relative to the prototype is represented by dots, the dashes represents regions without sequence information. 1: nt 1311–1322, a 63 bp insertion of GCGCCATGAGACTGAAACACCATGTAGTCAGTATAGTGGTGGAAGTGGGGGTGGTTGCAGTCA; 2: nt 4192–4193, a 3 bp insertion of TTG; 3: nt 4196–4197, a 3 bp insertion of TTG; 4: nt 7772–7807, a 36 bp deletion of AACTAAATGTCACCCTAGTTCATACATGAACTGTGT.
(PDF)

Table S1 **Reference sequences used to perform the alignment for phylogenetic analysis.**
(PDF)
