# Supplementary material for: Human Papillomavirus 16 Non-European Variants Are Preferentially Associated with High-Grade Cervical Lesions
Source: PLoS One. 2014 Jul 1;9(7):e100746. doi: 10.1371/journal.pone.0100746 (PMC4077691; doi:10.1371/journal.pone.0100746)
Supplement: Table S1 — Reference sequences used to perform the alignment for phylogenetic analysis. (PDF) [file pone.0100746.s002.pdf]

Table S1. Reference sequences used to perform the alignment for phylogenetic analysis.

Type	Lineage	Sublineage	Variant Genome ID	GenBank Accession #	Other names*
HPV16	A	A1	Ref	K02718	European (E)
		A2	W0122	AF536179	E
		A3	AS411	HQ644236	E
		A4	W0724	AF534061	Asian, E(As)
	B	B1	W0236	AF536180	African-1, Afr1a
		B2	Z109	HQ644298	African-1, Afr1b
	C		R460	AF472509	African-2, Afr2a
	D	D1	QV00512	HQ644257	North American (NA)1
		D2	QV15321	AY686579	Asian-American (AA)2
		D3	QV00995	AF402678	Asian-American (AA)1
